# Development and evaluation of the modiolar research array – multi-centre collaborative study in human temporal bones

**DOI:** 10.1179/1754762811Y0000000007

**Published:** 2011-08

**Authors:** Robert J S Briggs, Michael Tykocinski, Roland Lazsig, Antje Aschendorff, Thomas Lenarz, Timo Stöver, Bernard Fraysse, Mathieu Marx, J Thomas Roland, Peter S Roland, Charles G Wright, Bruce J Gantz, James F Patrick, Frank Risi

**Affiliations:** 1 University of Melbourne and HEARing CRC, Melbourne, Australia; 2 Universität Freiburg, Germany; 3 Medizinische Hochschule Hannover, Germany; 4 Hôpital Purpan, Toulouse, France; 5 New York University, USA; 6 University of Texas, Southwestern, USA; 7 The University of Iowa Carver College of Medicine, USA; 8 Cochlear Limited, Sydney, Australia

**Keywords:** Cochlear implant, Temporal bone, Electrode, Perimodiolar, Cochleostomy, Round window, Hearing preservation

## Abstract

**Objective:**

Multi-centre collaborative study to develop and refine the design of a prototype thin perimodiolar cochlear implant electrode array and to assess feasibility for use in human subjects.

**Study Design:**

Multi-centre temporal bone insertion studies.

**Materials and Methods:**

The modiolar research array (MRA) is a thin pre-curved electrode that is held straight for initial insertion with an external sheath rather than an internal stylet. Between November 2006 and February 2009, six iterations of electrode design were studied in 21 separate insertion studies in which 140 electrode insertions were performed in 85 human temporal bones by 12 surgeons. These studies aimed at addressing four fundamental questions related to the electrode concept, being: (1) Could a sheath result in additional intra-cochlear trauma? (2) Could a sheath accommodate variations in cochlea size and anatomies? (3) Could a sheath be inserted via the round window? and (4) Could a sheath be safely removed once the electrode had been inserted? These questions were investigated within these studies using a number of evaluation techniques, including X-ray and microfluoroscopy, acrylic fixation and temporal bone histologic sectioning, temporal bone microdissection of cochlear structures with electrode visualization, rotational tomography, and insertion force analysis.

**Results:**

Frequent examples of electrode rotation and tip fold-over were demonstrated with the initial designs. This was typically caused by excessive curvature of the electrode tip, and also difficulty in handling of the electrode and sheath. The degree of tip curvature was progressively relaxed in subsequent versions with a corresponding reduction in the frequency of tip fold-over. Modifications to the sheath facilitated electrode insertion and sheath removal. Insertion studies with the final MRA design demonstrated minimal trauma, excellent perimodiolar placement, and very small electrode dimensions within scala tympani. Force measurements in temporal bones demonstrated negligible force on cochlear structures with angular insertion depths of between 390 and 450°.

**Conclusion:**

The MRA is a novel, very thin perimodiolar prototype electrode array that has been developed using a systematic collaborative approach. The different evaluation techniques employed by the investigators contributed to the early identification of issues and generation of solutions. Regarding the four fundamental questions related to the electrode concept, the studies demonstrated that (1) the sheath did not result in additional intra-cochlear trauma; (2) the sheath could accommodate variations in cochlea size and anatomies; (3) the sheath was more successfully inserted via a cochleostomy than via the round window; and (4) the sheath could be safely removed once the electrode had been inserted.

## Introduction

The benefits of preserving residual hearing at the time of cochlear implantation to allow combined electric and acoustic stimulation have been well demonstrated (von Ilberg *et al*., 1999; Gantz *et al*., 2005; James *et al*., 2005; Lenarz *et al*., 2006; Skarzynski *et al*., 2006; Büchner *et al*., 2009). The Advance Off-Stylet™ insertion technique with perimodiolar placement of an intra-cochlear electrode into scala tympani has also been demonstrated to reduce intra-cochlear trauma (Roland, 2005) and improve clinical outcomes (Aschendorff *et al*., 2007; Skinner *et al*., 2007). Deep insertion of a straight electrode results in significant impact on the lateral cochlear wall and hence basilar membrane. This has been demonstrated to cause rupture of the basilar membrane and can result in electrode displacement into scala vestibuli in some cases (Adunka and Kiefer, 2006; Finley *et al*., 2008). Clinical studies with both 10 mm (Gantz and Turner, 2004) and 16 mm (Lenarz *et al*., 2006; Büchner *et al*., 2009) hearing preservation straight electrodes manufactured by Cochlear Ltd, have achieved good hearing preservation both with cochleostomy and round window insertions. The shallower angular insertion depth of these electrodes (approximately 180–280°) means that the outcome with electric stimulation alone is potentially limited compared to the Contour Advance™ electrode, which typically achieves an insertion depth of 390–450° with excellent perimodiolar position when the Advance Off-Stylet insertion technique is used.

The Contour Advance electrode has a diameter of 0.5–0.8 mm due to the silicone needed to envelop the internal platinum stylet. This means that a 1.2–1.5 mm cochleostomy is required. Clinical experience has demonstrated that while residual hearing can be preserved using the Contour Advance electrode, there is an average sensorineural hearing level drop of 25 dB or more, and total hearing loss in some cases (Fraysse *et al*., 2006). In many cases, this additional hearing loss means that a hearing aid is no longer of benefit in the implanted ear. Furthermore, the Advance Off-Stylet technique is not used consistently by all surgeons when inserting the Contour Advance electrode.

In order to improve on the hearing preservation results achieved by the Contour Advance, a much smaller diameter pre-curved electrode that can be implanted using a consistent surgical technique is required. This would allow a smaller cochleostomy, or even allow for insertion through the round window, avoiding lateral wall trauma and ensuring consistent placement within scala tympani.

The modiolar research array (MRA) is a prototype thin, pre-curved array that is held straight prior to insertion by an external polymer sheath which is removed after full insertion of the array. The elimination of the internal stylet and surrounding silicone rubber reduces the electrode volume by up to 75%, resulting in a thin, flexible perimodiolar electrode with dimensions equivalent to current lateral wall electrodes that are designed to preserve residual hearing.

The concept of MRA was presented to the principal authors by Cochlear Ltd in November 2006 and a multi-centre collaborative study was initiated to develop the design and function of the MRA and to assess surgical usability and safety. Each author has extensive experience in the assessment of surgical usability and safety of intra-cochlear electrodes, and utilizes a variety of techniques to assess the dynamics of insertion, final electrode position, and to evaluate intra-cochlear trauma.

## Materials and methods

**Figure 1:**
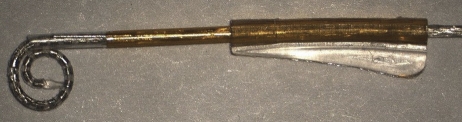
MRA (version 1), sheath was not removable, only retractable.

Initial experience with the first concept prototype MRA (Fig. 1) in plastic cochlear models demonstrated that use of an external sheath rather than an internal stylet to keep a pre-curved electrode straight for initial insertion was feasible. However, a number of questions were raised regarding the potential for intra-cochlear trauma and practicality of this concept clinically. These included: (1) Could a sheath result in additional intra-cochlear trauma? (2) Could a sheath accommodate variations in cochlea size and anatomies? (3) Could a sheath be inserted via the round window? and (4) Could a sheath be safely removed once the electrode had been inserted?

Methods to address these questions involved video recordings of microsurgical insertion to provide information regarding the handling of the electrode and function of the sheath. The trajectory and dynamics of intra-cochlear sheath and electrode movement during insertion was assessed using either microfocus fluoroscopy (Xu *et al*., 2009) or direct observation in microdissected human temporal bones (Wright and Roland, 2005). Final electrode position was assessed by imaging techniques that included plain film X-ray, high-resolution microfocus X-ray, computed tomography, or rotational tomography (Aschendorff *et al*., 2005). Electrode position relative to intra-cochlear structures and the presence of any intra-cochlear trauma was assessed by acrylic fixation and temporal bone histologic sectioning as well as the examination of microdissected specimens. Electrode insertion forces in temporal bones (Roland, 2005) were measured and the forces generated by the MRA electrode were compared with those generated by the Contour Advance and by straight lateral wall electrodes. These studies included a combination of both informal studies to explore design options/issues and formal studies to assess intra-cochlear trauma and insertion safety.

**Figure 2:**
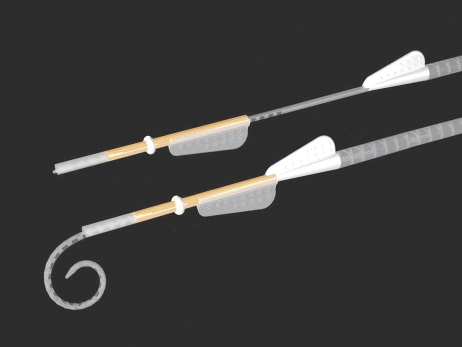
MRA electrode (version 6) loaded in sheath (top), and pre-curved, prior to loading, or after insertion prior to removal of sheath (bottom).

A number of design changes/improvements were implemented throughout this iterative process, resulting in six versions of the prototype MRA being tested, with MRA version 6 shown in Fig. 2.

MRA version 6 has an outer sheath which is split along the lateral surface allowing for removal of the sheath after implantation of the array. The sheath has a white doughnut-shaped stopper (1.2 mm diameter) that limits the insertion depth of the sheath for an Advance Off-Stylet style of insertion. The sheath diameter itself is only 0.65 mm, allowing for a cochleostomy size of 0.7–0.8 mm, or potentially for insertion through the round window. The intra-cochlear portion of the sheath has a soft tip to prevent potential trauma due to the sheath, while the remainder of the sheath is constructed from a more rigid polymer. Both the array and external sheath have wings for handling, to help orientate the array relative to the sheath, and for sheath removal. The array itself has an apical diameter of 0.3 mm and basal diameter of 0.5 mm (compared to 0.5 and 0.8 mm, respectively, for the Contour Advance). The design length of the MRA is currently similar to the Contour Advance, so 17 mm (modiolar distance from the tip of the array to opening of the cochlea) or approximately 390–450° angular insertion depth, with 22 half-band platinum electrode contacts.

**Figure 3:**
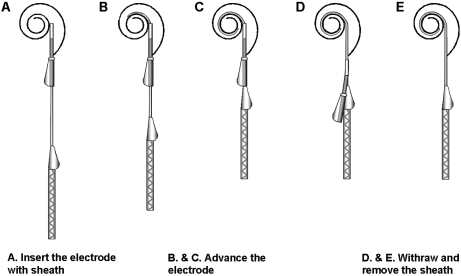
MRA (version 6) electrode insertion technique.

The proposed insertion technique for the MRA version 6 is illustrated in Fig. 3. The arrays used for the temporal bone studies were supplied ‘unloaded,’ i.e. with the array in its pre-curved shape. Prior to insertion the electrode is pulled back within the sheath (Fig. 3A) until the tip of the electrode is within the sheath. The sheath/electrode can then be inserted via a cochleostomy or round window to as far as the sheath stopper (Fig. 3B). At this point the sheath cannot be advanced further and so the array is advanced through the sheath until the white array handle contacts the sheath (Fig. 3C). The sheath is then removed by holding the electrode in position and pulling the sheath over the electrode handle (Fig. 3D) which is tapered to open the sheath and facilitate removal.

## Results

### Could a sheath result in additional intra-cochlear trauma?

**Figure 4:**
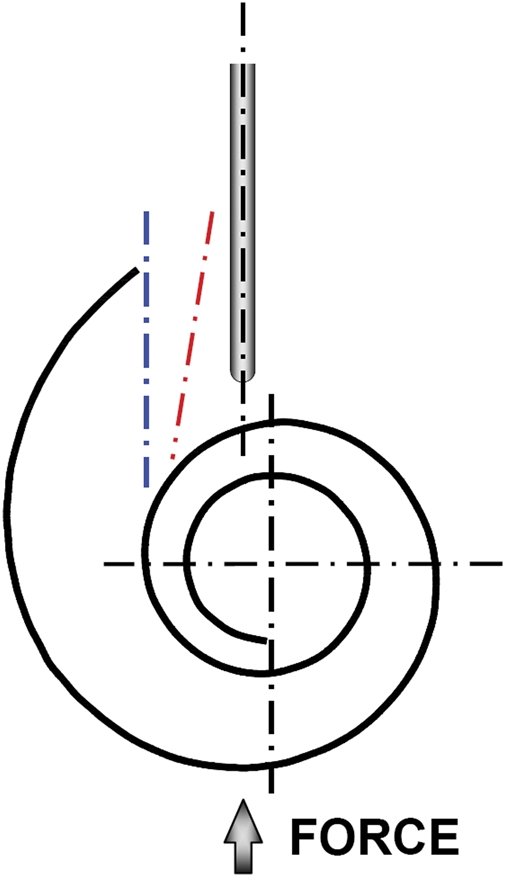
Sheath insertion force trajectory tested versus typical cochleostomy trajectory (blue) and round window trajectory (red).

The prototype sheath was designed with the distal portion of the sheath constructed from a soft silicone-like material to reduce the potential for intra-cochlear trauma from the sheath. To assess the potential for trauma, a comparative study of insertion forces using a ‘worst-case’ trajectory (Fig. 4) was conducted for the MRA as well as other intra-cochlear electrodes. A representative lubricated plastic model of the cochlea was used to compare forces during initial insertion. This ‘worst-case’ trajectory, with the axis of the electrode directed toward the modiolus with only a slight offset to the modiolar axis, will result in both an initial contact force and bending force of the tip of the electrode. Electrode insertion force testing as reported in the literature will typically use an ‘ideal’ cochleostomy or round window insertion trajectory (blue and red dashed lines, respectively, in Fig. 4) that may not adequately represent this potential initial contact force.

**Figure 5:**
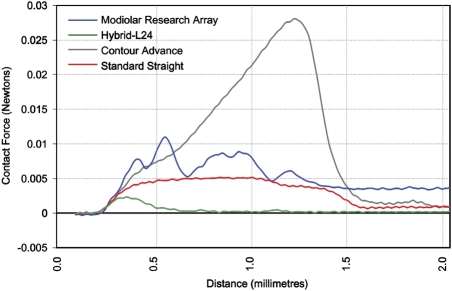
Modiolar contact force (N) measured for the MRA (sheath/electrode) versus Contour Advance, Nucleus Straight and Hybrid L24. Image courtesy of New York University.

Fig. 5 plots the mean insertion force in Newton's (N) measured in a plastic cochlea model using this ‘worst-case’ contact trajectory. The mean peak contact force of the MRA electrode (loaded within the sheath) is 0.013 N (*n* = 3, stdev = 0.002 N), compared to 0.006 N (*n* = 3, stdev = 0.0005 N) for the Nucleus^®^ Straight electrode, 0.003 N (*n* = 3, stdev = 0.0003 N) for the Hybrid™ L24 electrode, and 0.029 N (*n* = 3, stdev = 0.005 N) for the Contour Advance electrode. The maximum contact force for the MRA sheath therefore is not considered excessive and unlikely to result in any greater intra-cochlear (modiolar) trauma than current commercial electrode arrays, which have all been studied extensively and have not reported significant modiolar trauma.

Although not directly comparable, in terms of absolute force values the mean peak modiolar contact force of 0.013 N measured for the MRA sheath, or even 0.029 N for the Contour Advance, is still significantly less than the mean peak lateral wall force of 0.095 N reported using similar plastic models for Contour™ electrodes inserted using a standard insertion technique (Todd *et al*., 2007) or for mean peak lateral wall forces of between 0.13 and 0.35 N measured for straight electrodes (Adunka *et al*., 2004; Radeloff *et al*., 2009).

The potential for trauma as a result of incorrect orientation of the sheath with pre-curved array was also assessed in microdissected human temporal bones by orientating the array within the sheath toward the basilar membrane prior to advancing the array through the sheath. In each case the array corrected itself without dislocating any intra-cochlear membranes. Subsequent explantation of the arrays from the cochlea was also performed without identifiable trauma associated with removal of either the sheath or the electrode array.

In addition to the sheath contact force measurements and insertions into microdissected temporal bones, a total of 60 insertions (38 cochleostomy, 22 round windows) with MRA versions 1–5 were performed in 32 human temporal bones to assess both surgical usability and insertion trauma. Of these, 19 temporal bones (fresh frozen) were fixed and histologically evaluated to assess intra-cochlear trauma from either the sheath, or electrode insertion. Trauma was graded using a scale of 0–4 (Eshraghi *et al*., 2003). The definitions of the grading scale are detailed in Table 1, and the results are detailed in Table 2.

**Table 1:** Grading scale used to evaluate intra-cochlear trauma

Grade	Trauma
0	No trauma
1	Elevation of basilar membrane (BM)
2	Rupture of BM or spiral ligament (SL)
3	Dislocation into scala vestibuli (SV)
4	Fracture of osseous spiral lamina (OSL) or modiolus

**Table 2:** Evaluation of intra-cochlear trauma (MRA versions 1–5)

Sample	RW/C	Angular depth of insertion									
		0–45°	45–90°	90–135°	135–180°	180–225°	225–270°	270–315°	315–360°	360–405°	405–450°
1	C	0	0	0	0	0	1	0	0	–	–
2	C	0	0	0	0	0	0	0	0	–	–
3	C	0	0	0	0	0	0	0	0	0	–
4	C	0	0	0	0	0	0	0	0	0	–
5	C	0	0	0	0	0	0	0	0	–	–
6	C	0	0	0	0	0	0	0	0	0	–
7	C	0	0	0	0	0	0	0	0	0	–
8	C	0	0	0	0	1*	–	–	–	–	–
9	C	0	0	0	3*	–	–	–	–	–	–
10	C	0	0	0	0	0	0	0	0	0	–
11	C	0	0	0	0	0	0	0	0	0	–
12	C	0	0	0	0	0	0	0	0	0	–
13	RW	0	0	0	0	0	1	0	0	–	–
14	RW	0	0	0	3*	–	–	–	–	–	–
15	RW	0	0	0	0	0	0	0	0	0	–
16	RW	0	0	0	0	0	0	0	0		–
17	RW	0	0	0	0	0	0	0	0	0	–
18	RW	0	0	0	3*	–	–	–	–	–	–
19	RW	0	0	0	0	0	0	0	0	0	–

*Note:* *Tip fold-over.

**Figure 6:**
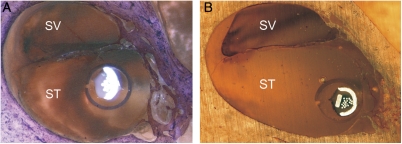
Mid-modiolar sections showing the electrode and sheath within scala tympani (A) cochleostomy insertion, CRC Hear, Melbourne, Australia. (B) round window insertion, Medizinische Hochschule Hannover, Germany. ST, scala tympani; SV, scala vestibuli.

There was no evidence of trauma to the modiolus and in general no evidence of intra-cochlear trauma due to either the sheath or electrode array. For the three specimens that did experience significant trauma (Grade 3), trauma was attributed to tip fold-over that occurred in each of the three specimens. In another specimen (Specimen 8) that also experienced a tip fold-over, the electrode remained in scala tympani. In another two of the specimens, one cochleostomy and one round window insertion, the sheath was left *in situ* after implantation to assess the position and orientation of the sheath and adjacent intra-cochlear structures, i.e. the sheath was not retracted post insertion of the electrode array. No trauma associated with the sheath was evident. Mid-modiolar sections of these two specimens show the distal tip of the sheath still visible in Fig. 6.

Acrylic fixation and sectioning of 17 human temporal bones implanted as part of a formal safety study with MRA version 6 was also undertaken. Trauma was graded using a scale of 0–4 according to Table 1, the results of which are detailed in Table 3.

**Table 3:** Evaluation of intra-cochlear trauma (MRA version 6)

Sample	RW/C	Angular depth of insertion									
		0–45°	45–90°	90–135°	135–180°	180–225°	225–270°	270–315°	315–360°	360–405°	405–450°
1	C	0	0	0	0	0	0	0	0	0	0
2	C	0	0	0	0	0	0	0	0	0	
3	C	3	0	0	0	0	0	0	0	0	0
4	C	0	0	0	0	0	0	0	0	0	–
5	C	0	0	0	0	0	0	0	0	–	
6	C	0	0	0	0	0	0	0	0	0	–
7	C	0	0	0	0	0	0	0	0	0	–
8	C	0	0	0	0	0	0	0	0	0	0
9	C	0	0	0	0	0	0	0	0	0	0
10	C	0	0	0	0	0	0	0	0	0	0
11	RW	0	0	0	0	0	0	0	0	–	–
12	RW	0	0	0	0	0	0	0	0	–	–
13	RW	0	0	0	0	0	0	0	0	0	–
14	RW	0	0	0	0	0	0	0	0	–	–
15	RW	0	0	0	0	0	0	0	0	0	–
16	RW	0	0	0	0	0	0	0	0	0	–
17	RW	0	0	0	0	0	0	0	0	–	–

This series included one specimen (Specimen 5) where at initial insertion the surgeon incorrectly oriented the array resulting in the array curving toward the lateral wall with a resultant tip fold-over. This was visualized on fluoroscopy and so it was elected to retract the electrode within the sheath and re-orientate. The electrode was then re-inserted successfully. Histological analysis of this temporal bone showed no evidence of intra-cochlear damage as a result of either the initial insertion and tip fold-over, or subsequent removal and re-insertion of the same electrode array.

**Figure 7:**
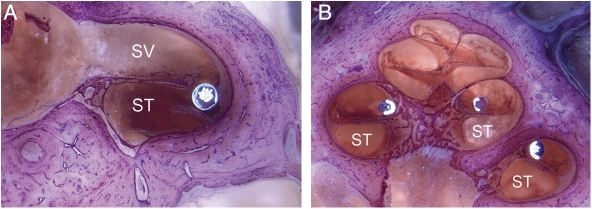
Showing Specimen 3 with (A) electrode entering scala vestibuli and (B) final perimodiolar position in scala vestibuli. Images courtesy of CRC Hear, Melbourne, Australia. ST, scala tympani; SV, scala vestibuli.

In another specimen (Specimen 3) it was found that the electrode passed through the cochleostomy into scala tympani but then immediately through the basilar membrane into scala vestibuli, where it then remained in a perimodiolar position (Fig. 7).

Clearly in this specimen, the trajectory of the electrode insertion had been in a too lateral direction resulting in insertion into scala vestibuli rather than scala tympani.

**Figure 8:**
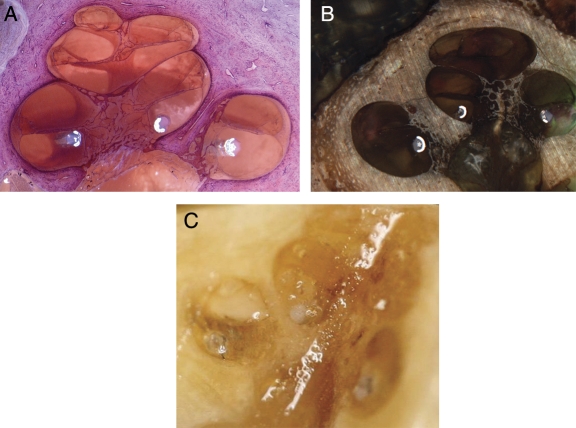
MRA (version 6) mid-modiolar section showing excellent modiolar placement and no evidence of trauma. Images courtesy of (A) CRC Hear, Melbourne, Australia; (B) Medizinische Hochschule Hannover; and (C) New York University.

Histological analysis of this series of human temporal bones with MRA version 6 confirmed excellent perimodiolar positioning with the electrode remaining in scala tympani and no evidence of trauma. Fig. 8 provides some typical examples of mid-modiolar sections with MRA version 6.

Histological evaluation in human temporal bones with MRA versions 1–5 and MRA version 6 verify that neither the sheath nor the electrode array result in significant intra-cochlear trauma, supporting the insertion force data.

Therefore, the use of a suitably designed straightening sheath, such as that used with the MRA, does not result in additional intra-cochlear trauma.

### Could a sheath accommodate variations in cochlea size and anatomies?

To control the insertion depth of the sheath prior to electrode insertion, a physical stopper consisting of a 1.2 mm diameter white silicone doughnut attached to the body of the sheath was used. This provided an absolute insertion depth for the sheath. The basal cochlear dimensions can however vary in size, and there is also the consideration of the insertion site (round window or cochleostomy). One important design aspect was to identify whether a single sheath could accommodate variations in cochlear size and insertion site.

**Figure 9:**
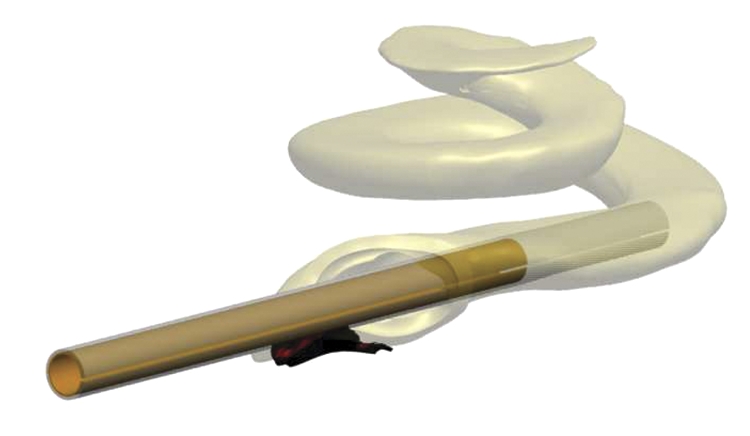
Three-dimensional model of the scala tympani with sheath inserted via an inferior-anterior cochleostomy.

A three-dimensional computer model of the cochlea, including scala tympani, scala vestibuli and the round window anatomy, was generated by stacking digitized two-dimensional histological slices from a single human temporal bone. Fig. 9 shows the three-dimensional model of the scala tympani only with sheath inserted via an inferior-anterior cochleostomy.

**Figure 10:**
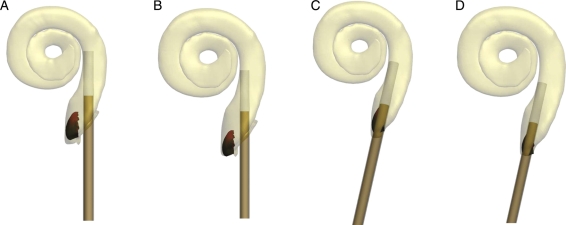
Examples of small and large cochlea with equal intra-cochlear sheaths lengths positioned with cochleostomy (A, B) or round window (C, D) insertion points and trajectories showing the final distal sheath location for each.

The review of cochlea size and morphology (Escudé *et al*., 2006) was used to scale the computer model such that representative ‘small’ and ‘large’ cochlea were generated. Insertion trajectories for both round window and inferior-anterior cochleostomy locations were then modeled to determine an optimal distal sheath location within scala tympani under a number of different conditions. The combination of a ‘small’ cochlea with a cochleostomy insertion/trajectory provided a theoretical worst case for sheath over-insertion (Fig. 10A), and alternatively the combination of a ‘large’ cochlea with round window insertion/trajectory provided a theoretical worst case for sheath under-insertion (Fig. 10D).

Various intra-cochlear sheath lengths were modeled and a single intra-cochlear sheath length identified that could accommodate the reported variations in cochlea size. This sheath length was then tested in vitro both in plastic cochlea models and also in human temporal bones.

**Figure 11:**
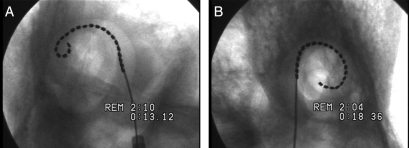
MRA (version 2) showing (A) tip fold-over (B) full insertion only after electrode contacts lateral wall. Images courtesy of CRC Hear, Melbourne, Australia.

**Figure 12:**
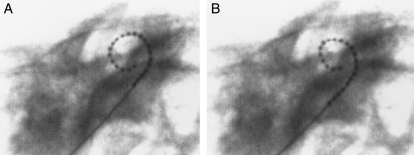
MRA (version 4) showing (A) over-insertion and (B) the same insertion after the electrode is pulled back. Images courtesy of New York University.

**Figure 13:**
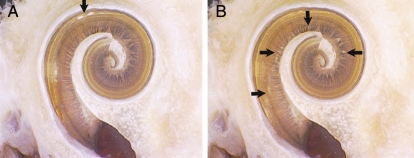
MRA (version 5) showing (A) sheath and array inserted and contact with the lateral wall and (B) after contact and full insertion with sheath removed. Images courtesy of UT Southwestern Medical Centre.

**Figure 14:**
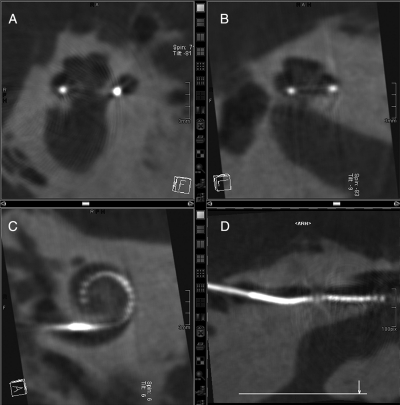
MRA (version 5) rotational tomography following human temporal bone insertion: (A and B) different mid-modiolar views, (C) transcochlear view, and (D) curved reformation of the MRA array located in the basal scala tympani. Images courtesy of University of Freiburg, Germany.

Fluoroscopic imaging of temporal bones during electrode insertion, insertion in microdissected temporal bones, and computed tomography imaging of implanted temporal bones all indicated that a single sheath length could accommodate anatomical variations. These studies did however highlight that distal pre-curvature of the array was too acute in earlier versions of the MRA resulting in either tip fold-over beyond the first turn, or in the tip meeting resistance at approximately 270–360° angular insertion depth. In the latter, further insertion resulted in the proximal array contacting the lateral wall, providing the additional force required to overcome resistance at the tip, and only then was a full insertion of 390–450° achieved with good perimodiolar position (Figs 11–14).

The curvature of the distal array was relaxed iteratively in each of the MRA versions 1–6. Insertion dynamics and the incidence of tip fold-overs were found to progressively improve. Further relaxation of the distal curvature was achieved by reducing the intra-cochlear array length (modiolar wall length) from 19 to 17.5 mm, equating to an approximate 30° reduction in the intended design depth of 420–450 to 390–420°.

**Figure 15:**
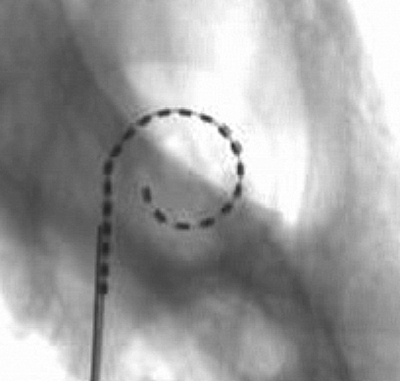
MRA (version 6) microfocus fluoroscopy showing excellent final positioning and depth of the array. Images courtesy of CRC Hear, Melbourne, Australia.

Insertions with MRA (version 6) performed under fluoroscopy showed significant overall improvement in insertion dynamics compared to previous versions. The sheath length and start point for an advanced through sheath insertion followed a smooth modiolar trajectory to the intended insertion depth without lateral wall contact and with excellent final positioning and depth of the array (Fig. 15).

The results of modeling and subsequent studies in human temporal bones using fluoroscopic imaging to examine sheath/electrode dynamics therefore supports the hypothesis that a single sheath length can accommodate variations in normal cochlea anatomy and also variations in insertion sites.

### Could a sheath be inserted via the round window?

The sheath diameter of 0.65 mm and non-rigid construction of the distal sheath are such that insertion via the round window should be feasible. This hypothesis was tested for versions 1–5, with a total of 22 insertions in human temporal bones via the round window.

Round window insertions were typically more challenging with the tip fold-over rate being 2–3 times that for insertions via a standard inferior-anterior cochleostomy. The trajectory of the array through the round window is one potential contributing factor, potentially distorting the sheath. Also with MRA version 5, it was noted that in some cases removal of the sheath in round window specimens required more force than cochleostomy specimens. MRA version 5 was the first version to include the split sheath, allowing for complete removal of the sheath post insertion. The greater effort required to remove the sheath in these cases may have been attributed to a reduction in the diameter of the split sheath due to its trajectory through the round window. The resulting compression of the split sheath could potentially then clamp the electrode array resulting in increased friction with a greater force then being required to remove the sheath.

Although the results show that the MRA can successfully be inserted via the round window, the incidence for surgical complication and tip fold-over was shown to be 2–3 times greater than insertions via an inferior cochleostomy.

### Could a sheath be safely removed once the electrode had been inserted?

MRA versions 1 through 4 included a sheath design that was retractable, but not removable, i.e. the sheath could be pulled back along the array such that its final position was extra-cochlea; however, it remained attached to the proximal electrode lead. While this design was adequate for the purpose of temporal bone studies to assess intra-cochlear electrode dynamics and trauma, it would not be suitable for clinical use. MRA version 5 included a removable sheath, allowing for the retraction and complete removal of the sheath. Removal of the sheath was achieved by an axial split along the lateral surface of the sheath. The silicone electrode wing was modified to form a tapered ramp which (on retraction of the sheath) opened and guided the sheath superiorly away from the electrode array until the sheath was completely removed (Fig. 3).

A total of 25 insertions with MRA version 5 were performed using 19 human temporal bones. The performance of the removable sheath was assessed by the investigators as adequate. In general the use of more robust forceps, rather than the fine tipped forces typically used to grip the silicone wings in this study, was identified as a potential area for improving sheath removal. Minor modifications to the shape and softness of the wings were also identified.

The split sheath design as implemented and tested on MRA version 5 was identified as adequate. Removal of the sheath could be completed safely and without affecting the implanted electrode array.

## Discussion

The development of new and novel electrodes is essential in order to continue and build on the excellent benefits already provided by the cochlear implant. The advantages of reducing trauma associated with electrode insertion are well understood and becoming more important as we implant patients with increasing levels of residual hearing. Current lateral wall hearing preservation electrodes generally require some trade-off between preserving residual hearing and having an adequate depth of insertion for electrical stimulation. The MRA is a novel approach that could potentially achieve both these goals.

Collaboration between manufacturer and surgeon in the field of electrode development is vital to ensure that such developments are safe and appropriate for wider clinical use. The development of the MRA highlights the advantages of involving multiple centres and a larger cohort of surgeons each having a unique experience in the field of assessing safety of intra-cochlear electrodes. This approach has facilitated the development of a new and novel approach to atraumatic placement of a very thin and flexible perimodiolar array. The current MRA requires a cochleostomy of only 0.7–0.8 mm in diameter, compared to the 1.0–1.5 mm diameters typically required for current commercial electrodes, which may be a contributor to hearing loss related to surgery (Fraysse *et al*., 2006). The rate of either tip fold-over or dislocation into scala vestibuli for round window insertions was 2–3 times that for insertions via a standard inferior-anterior cochleostomy. Although we have identified that round window insertions with the current MRA are not recommended, there is ongoing development to optimize the design so as to be compatible with round window insertions.

Results with early MRA prototypes identified a high incidence of tip fold-over. Iterative improvements to the MRA design produced a final version in which this problem was almost completely resolved. The investigators acknowledge that a proportion of the fold-overs were attributable to issues with surgical handling due to the continued interdependence between the sheath and the array. Surgeons' using the MRA for the first time typically experienced a rate of tip fold-over that was three times the rate of those with prior experience. Therefore, experience and familiarity with the current MRA design does translate to more reliable electrode placement; however, the risks are still present. One critical recommendation to the manufacturer for future versions of the MRA is to remove the current interdependence between the sheath and the array so that the angular orientation and linear translation of the array relative to the sheath is not dependent on the surgeons handling of the array.

The concept of a sheath, or ‘inserter’ was initially investigated by both the Bionic Ear Institute and Advanced Bionics Corporation in the late 1990s. These concepts, however, were abandoned due to the rigidity and size of the sheaths being investigated and resultant trauma. This was also a concern with the MRA concept when presented to the authors originally; however, initial human temporal bone feasibility studies and intra-cochlear force studies with the MRA electrode highlighted the lack of intra-cochlear trauma and considerably lower intra-cochlear contact forces than stylet-based electrodes, which was a very promising result. Although the initial intra-cochlear contact forces measured were greater than those for straight lateral wall electrodes, such as the Hybrid L24, it was demonstrated that the use of a sheath could potentially improve hearing preservation outcomes when compared with the Contour Advance electrode, for example.

The size and insertion dynamics of the MRA result in a potentially atraumatic electrode taking up minimal volume within scala tympani with insertion to 390–420° (equivalent to a 25 mm lateral wall insertion depth) with minimal intra-cochlear contact and lateral wall forces. These reduced forces and contact pressures, which in lateral wall electrode accumulate and increase significantly beyond 20 mm, are not present with the MRA, allowing for consistently greater depth of insertion with a reduced potential for trauma.

## Conclusion

The MRA is a novel, very thin perimodiolar prototype electrode array that has been developed using a systematic collaborative approach. The different evaluation techniques employed by the investigators contributed to the early identification of issues and generation of solutions. Regarding the four fundamental questions related to the electrode concept, the studies demonstrated that (1) the sheath did not result in additional intra-cochlear trauma; (2) the sheath could accommodate variations in cochlea size and anatomies; (3) the sheath was more successfully inserted via a cochleostomy than via the round window; and (4) the sheath could be safely removed once the electrode had been inserted.
